# Supplementary figures and images for: A High-Performance Liquid Chromatography—Mass Spectrometry Method for Simultaneous Determination of Vancomycin, Meropenem, and Valproate in Patients with Post-Craniotomy Infection
Source: Molecules. 2023 Mar 7;28(6):2439. doi: 10.3390/molecules28062439 (PMC10051502; doi:10.3390/molecules28062439)

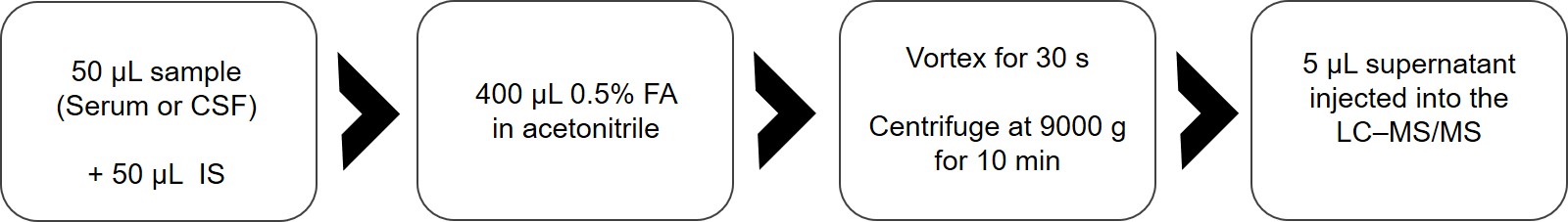

Supplement: Supplementary file 1 [file molecules-28-02439-s001.zip › molecules-2128657-supplementary.jpg]
